# Computational and experimental investigations of the giant dielectric property of Na_1/2_Y_1/2_Cu_3_Ti_4_O_12_ ceramics

**DOI:** 10.1038/s41598-023-31879-z

**Published:** 2023-03-21

**Authors:** Jakkree Boonlakhorn, Punpatsorn Suksangrat, Weerachai Sarakorn, Sriprajak Krongsuk, Prasit Thongbai, Pornjuk Srepusharawoot

**Affiliations:** 1 Department of Basic Science and Mathematics, Faculty of Science, Thaksin University, Songkhla Campus, Songkhla, 90000 Thailand; 2 Giant Dielectric and Computational Design Research Group (GD–CDR), Department of Physics, Faculty of Science, Khon Kaen University, Khon Kaen, 40002 Thailand; 3 Department of Mathematics, Faculty of Science, Khon Kaen University, Khon Kaen, 40002 Thailand; 4 Institute of Nanomaterials Research and Innovation for Energy (IN-RIE), Khon Kaen University, Khon Kaen, 40002 Thailand

**Keywords:** Electronic devices, Electronic structure, Ceramics

## Abstract

A modified sol-gel method was used to successfully produce Na_1/2_Y_1/2_Cu_3_Ti_4_O_12_ ceramics with high dielectric permittivity. The dielectric permittivity of Na_1/2_Y_1/2_Cu_3_Ti_4_O_12_ ceramics reaches values larger than 10^4^ at room temperature and 1 kHz. Moreover, these ceramics exhibit two distinct thermally induced dielectric relaxations over a broad temperature range. The loss tangent is indeed small, ~0.032–0.035. At low temperatures, dielectric relaxation was attributed to the oxygen vacancy effect, while at high temperatures, it was attributed to grain boundary and sample-electrode contact effects. Our calculations revealed that Y and Na ions are likely to occupy Ca and Cu sites, respectively. As a result, other Cu related phases, especially CuO, were observed at the grain boundaries. Based on our analysis, there is a charge compensation between Na and Y ions in Na_1/2_Y_1/2_Cu_3_Ti_4_O_12_. Additionally, the Cu^+^ and Ti^3+^ states observed in our XPS study originate from the presence of an oxygen vacancy in the lattice. Last, the primary cause of the enormous dielectric permittivity of Na_1/2_Y_1/2_Cu_3_Ti_4_O_12_ ceramics primarily comes from the internal barrier layer capacitor effect.

## Introduction

People rely heavily on high-performance electronic devices in their everyday lives. Numerous electronic innovations have therefore been developed, beginning with enhancement of the electronic properties of materials used in essential components and ending with the production of electronic devices. The most often expressed viewpoint is the desire to shrink the size of devices while increasing their performance^1–5^. Another perspective is the need to decrease the use of hazardous compounds in electronic devices^5–12^. In recent years, high-tech devices, particularly ones used for electric power storage, have been extensively discussed^1–4^. Previously published studies indicated that the performance of capacitors is enhanced since their use is critical for temporary electric storage^1–5^. Ceramic capacitors are widely used as essential components in a variety of devices, such as graphics cards and random-access memory (RAM)^13^. The dielectric properties of a material determine its suitability for various applications, especially for capacitors. Dielectric constants (ε′) and dielectric loss tangents (tan δ) are critical parameters indicating the dielectric performance of materials^14^. Recent interest in metal ion co-doped TiO_2_, metal ion co-doped SnO_2_, and undoped, single doped, and co-doped ACu_3_Ti_4_O_12_ (A=Ca, Cd, Na_1/2_Y_1/2_, Sm_2/3_, Y_2/3_) ceramics have caught the attention of academics interested in investigating their structural and dielectric properties^1–6,15–22^. Na_1/2_Y_1/2_Cu_3_Ti_4_O_12_ (NYCTO) is one of the most popular ceramic dielectrics studied in recent years^18–20,22^.

For NYCTO ceramics, the two most prominent areas of investigation are enhancing its dielectric properties and investigating the causes of its colossal dielectric response^18–20,22^. In general, the high ε′ of NYCTO ceramics is interesting. According to the internal barrier layer capacitor (IBLC) model, the most widely recognized origin of the high ε′ of NYCTO and similar ceramics is interfacial polarization^23,24^. This is due to the heterogeneity in their microstructure. Advanced technological methods have shown the presence of semiconducting grains and insulating grain boundaries (GBs) in NYCTO and related ceramics^18–20,22–24^. From their microscopic scale based on an IBLC model, the nanoscale barrier layer capacitance (NBLC) mechanism and domain boundary impact comes from intrinsic defects. They have been found to be the origins of the gigantic dielectric response in NYCTO^25,26^. Our earlier work showed that NYCTO ceramics produced through a solid-state reaction (SSR) and sintered at 1100 °C for various times attained high ε′ values, 0.13−2.30×10^4^, with low tanδ values, 0.030−0.111^18^. Ahmad and Kotb reported a reduced sintering temperature *via* the use spark plasma (SP) sintering. They discovered a high ε′ of approximately 2.49×10^4^ in an NYCTO ceramic sintered at 975 °C for 10 minutes. Nevertheless, its tanδ remains very high (~3.39)^20^. Additionally, Kotb and Ahmad revealed that a ε′ value of 4.50×10^3^ and a loss tangent of 0.055 can be obtained in an NYCTO ceramic produced using a SSR and sintered in air for 10 h at 1050 °C^19^. After the SSR, an ε′ value of more than 10^4^ with a tanδ of less than 0.10 was obtained using a high sintering temperature (1100 °C). Wet chemical techniques, specifically a modified sol-gel technique, have been proposed as viable manufacturing methods for producing dielectric ceramics with desirable properties *via* low-temperature sintering^6,9,10,12^. While the dielectric properties of ACu_3_Ti_4_O_12_ ceramics produced through a wet chemical method have been extensively documented^6–12^, they have never been published for NYCTO. In a few NYCTO studies, minor decomposition of additional phases was observed in SEM images^18,20,27^. However, XRD cannot identify them. These phases may generate high ε′ values with low tanδ values in NYCTO. As a result, a modified sol-gel method should be used to prepare NYCTO. Although investigations of NYCTO ceramics^18–20,22^ have been extensively reported, only experimental results have been presented. It is reasonable to combine both experimental and computational methods based on density functional theory (DFT) to gain insight into the electrical and dielectric properties of this ceramic.

NYCTO ceramics were effectively produced in this study using a modified sol-gel technique. Additionally, the structural and dielectric properties of NYCTO ceramics were systematically studied and evaluated. DFT calculations were performed to identify the lattice’s most stable position for Na, Y, Cu, Ti, and O atoms. All experimental and computational findings are discussed and compared to values published in the literature. The fabrication process is described from start to finish in the results and discussion.

## Discussion

**Figure 1:**
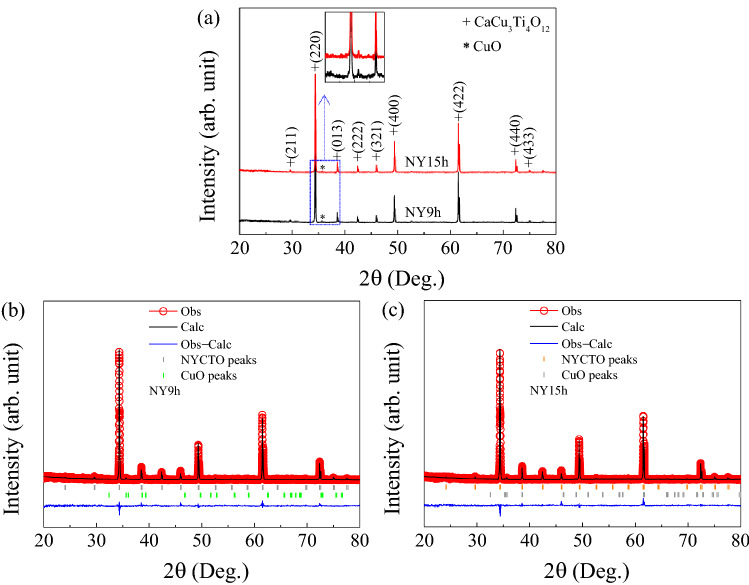
(**a**) XRD patterns of NY9h and NY15h ceramics. (**b**,**c**) respective Rietveld profile fits of these two samples.

The XRD patterns of NY9h and NY15h ceramics are shown in Fig. 1a. Analyses of the phase compositions showed a mixing of the CCTO-like phase with a small phase of CuO (ICSD No. 01−080−0076), which is seen at 2θ = 35.6. XRD data revealed that the CCTO-like phase exhibits a body-centered cubic structure possessing Im-3 (204) space group^28^. In our Rietveld refinement, the space group and atomic positions of CaCu_3_Ti_4_O_12_ (ICSD No. 01-075-2188) are used as initial parameters. We subsequently substituted Na and Y for 0.5 moles at Ca sites of this structure, known as the NYCTO structure. Atomic positions of Na, Y, Cu, Ti and O, occupation, and space group of NY9h and NY15h ceramics are given in Table 1. By using the parameters given in Table 1, the Rietveld technique was used to examine the XRD spectra of NY9h and NY15h ceramics. Rietveld profile fits for these two ceramic samples are shown in Fig. 1b,c. In these figures, it is demonstrated that the Rietveld approach could be used to effectively match the experimental data. Parameters indicating acceptable results of the Rietveld refinement were considered. For this, the *R*-factors for Rietveld refinement should be less than 10%^29^. Furthermore, the goodness of fit (GOF) coefficients should be lower than a value of 4. As shown in Table 2, the Rietveld technique produced suitable values for the weighted profile *R* factor (*R*_wt_), the expected profile *R* factor (*R*_exp_), the profile *R* factor (*R*_p_), and the GOF factors. The estimated lattice parameters (*ɑ*) of NY9h and NY15h ceramics were found to be 7.3816(0) and 7.3823(0) Å, respectively. Errors from all fitted XRD patterns were determined. It was found that they are indeed small, less than 10^−5^ Å. Hence, the errors of the lattice parameter are negligible in the current work. These results can be compared to those of published studies^5,10,12,28^. Additionally, the theoretical densities (ρ_T_) of NY9h and NY15h ceramics were estimated using the XRD spectra.

**Table 1:** Atomic positions of Na, Y, Cu, Ti and O, occupation, and space group of the NY9h and NY15h ceramics.

Samples	Atoms	Sites	Positions			Occupations	Space group
			x	y	z		
NY9h	Na	2a	0.0000	0.0000	0.0000	0.5000	Im-3 (204)
	Y	2a	0.0000	0.0000	0.0000	0.5000	
	Cu	6b	0.0000	0.5000	0.5000	1.0000	
	Ti	8c	0.2500	0.2500	0.2500	1.0000	
	O	24g	0.0000	0.1798	0.3026	1.0000	
NY15h	Na	2a	0.0000	0.0000	0.0000	0.5000	Im-3 (204)
	Y	2a	0.0000	0.0000	0.0000	0.5000	
	Cu	6b	0.0000	0.5000	0.5000	1.0000	
	Ti	8c	0.2500	0.2500	0.2500	1.0000	
	O	24g	0.0000	0.1798	0.3026	1.0000

**Table 2:** Structural data obtained from the Rietveld refinement, theoretical density (ρ_T_), and mean grain size (G) for the NY9h and NY15h ceramics.

Samples	NY9h	NY15h
*ɑ* (Å)	7.3816(0)	7.3823(0)
*R*_exp_ (%)	4.3501	4.4465
*R*_p_ (%)	4.2859	4.3088
*R*_wt_ (%)	5.5757	5.6276
GOF	1.6429	1.6018
ρ_T_ (g/cm^3^)	5.2025	5.2011
*G* (µm)	7.75±3.21	10.07±3.53

Based on the results, they were determined to be 5.2025 and 5.2011 g/cm^3^, respectively. It’s interesting to note that ρ_T_ in the NYCTO system is higher than those estimated in the CCTO system. From our synthesis procedures, 3 moles of CuO is initially used. According to our Rietveld refinement, it was found that there still remains a small percentage of 2.5 of CuO in our samples. Hence, this remaining CuO corresponds to 0.075 mole. The composition of our samples without the CuO phase should be Na_0.5_Y_0.5_Cu_3-0.075_Ti_4_O_12_ or Na_0.5_Y_0.5_Cu_2.925_Ti_4_O_12_.

**Figure 2:**
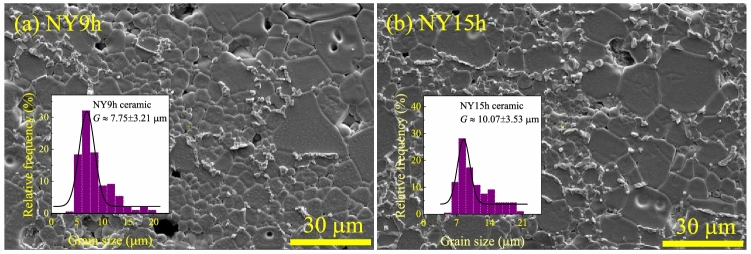
Polished cross-sectional SEM images and size distributions of (**a**) NY9h and (**b**) NY15h ceramics.

SEM images of the polished cross-sectional microstructure and size distribution of NY9h and NY15h ceramics after thermal treatment are shown in Fig. 2a,b. Different grain sizes are observed in these samples. Additionally, a small number of pores can be detected in both the NY9h and NY15h ceramics. Grain growth was found to be abnormal. A line intercept technique was used to determine the mean grain size of all samples. These results are shown in Table 2. It was found that the mean grain size of the NY9h ceramic is about 7.75 μm, which is smaller than that of the NY15h ceramic (∼10.07 μm).

This might be due to the longer sintering time of the NY15h ceramic. At the same sintering temperature, the average grain of the NY9h and NY15h ceramics is slightly larger than that of the NYCTO ceramics produced using an SSR^19^. Moreover, the microstructure of our specimens is particularly finer than that of the NYCTO ceramic fabricated by an SSR process and sintered at 1090 °C for 5 h. Furthermore, as shown at the GB layers, it is probable that a second phase was present. When SEM images and XRD findings are compared, it is plausible that a small secondary phase was present at GBs that may represent CuO.

**Figure 3:**
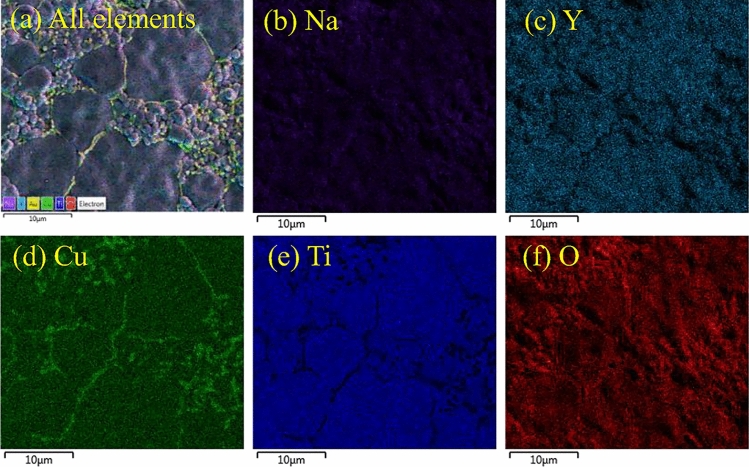
EDS mapping of the NY9h ceramic. Distribution of (**a**) all elements, (**b**) Na, (**c**) Y, (**d**) Cu, (**e**) Ti and (**f**) O elements in the NY9h sample.

As shown in Fig. 3, EDS mapping indicates that Na and Y elements are uniformly distributed on the ceramic surfaces. Ti ions are prevalent in the grain region, whereas they are scarce at the GB. Additionally, Cu-rich phases are also abundant in the GB layers (brighter zones). Based on these observations, CuO was found at the GBs. Before fabrication, the stoichiometric quantities and charge balance of the ceramics were carefully examined. Nevertheless, CuO may still have decomposed to form a second phase. According to our study, the presence of a related Cu phase in NYCTO is comparable to that described in the literature for this material^18,20^. CuO phase decomposition may be abnormal. Thus, a thorough investigation to determine the origin of the observed CuO phase decomposition should be carried. The bulk densities (ρ_B_) of the NY9h and NY15h ceramics were investigated using the Archimedes method and were found to be 4.5466 and 4.6514 g/cm^3^, respectively. According to the values of ρ_T_ and ρ_B_, the D factors for NY9h and NY15h were 87.39 and 89.43%, respectively.

**Figure 4:**
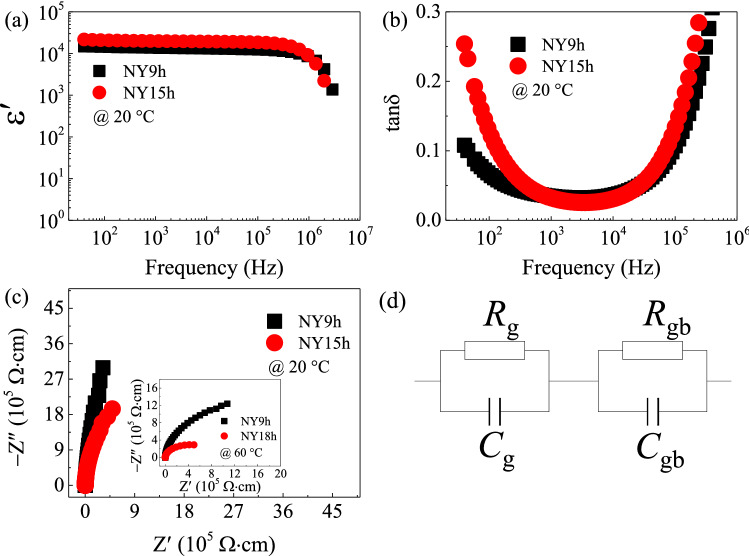
(**a**) Frequency dependencies of ε′ at 20 °C of NY9h and NY15h ceramics. (**b**) Frequency dependence of tan δ. (**c**) Impedance complex (Z*) plots at 20 °C; its inset shows Z* at 60 °C. (**d**) Equivalent circuit represents the electrical heterogeneous microstructure of semiconducting grain and insulating grain boundary.

We conducted a comprehensive investigation of the electrical and dielectric properties of sintered NYCTO ceramics. The results are expressed in terms of relative capacitance (C_p_) and tanδ during the measurements. The ε′ value can be determined using Eq. (1):

1 $$\varepsilon^{\prime} = \frac{{C_{p} d}}{{\varepsilon_{0} A}},$$

where d denotes the thickness of the sample, A is the area of the metal electrode, and ε_0_ represents the permittivity of empty space (ε_0_=8.85410^−12^ F/m). An impedance complex (Z*=Z′-jZ″) plot is used in dielectric research to explain the electrical properties of materials. To analyze the impedance data, the Z* plots were modeled by an ideal equivalent circuit of two parallel RC elements displayed in Fig. 4d. From Fig. 4d, the first RC element representing the grain response is connected in series with the second RC element indicating the grain boundary response. Equation (2) can be used to calculate the Z* plot:

2 $$Z^* = Z^{\prime} - jZ^{\prime\prime} = \frac{1}{{j\omega C_{0} \varepsilon^{ * } }},$$

where ω=2π*f* is the angular frequency, *C*_0_=ε_0_A/d is the capacitance of space. ε* denotes the complex dielectric permittivity, which is made up of a real part (ε′) or dielectric permittivity and an imaginary part (ε″=ε′tanδ) or total loss factor. The dielectric properties of NY9h and NY15h ceramics were investigated at various frequencies and temperatures in this study. The frequency dependencies of ε′ and tanδ in NY9h and NY15h ceramics are shown in Fig. 4a and b, respectively.

As demonstrated in these figures, the ε′ value of the NY15h ceramic is greater than that of the NY9h ceramic in the frequency range below 10^5^ Hz. According to SEM imagery, the rise in ε′ is strongly related to the increased grain size caused by the longer sintering time for the NY15h specimen, which indicates the IBLC behavior in the material. However, this microstructural component may not be the sole factor that significantly influences changes in the dielectric response. The ε′ values at 1 kHz of NY9h and NY15h ceramics were 1.37×10^4^ and 1.99×10^4^, respectively. Surprisingly, these two ceramics can reach ε′ values of more than 10^4^ by sintering at 1050 °C, while NYCTO ceramics produced using the SSR must be sintered at 1100 °C to achieve this value^18^. NYCTO ceramics produced utilizing a modified sol-gel technique can have good dielectric properties. As shown in Fig. 4b, in the frequency range below 10^3^ Hz, the tanδ of the NY15h ceramic is higher than that of the NY9h ceramic. Strong increases in tanδ, corresponding to a rapid decrease in ε′, were observed at frequencies higher than 10^5^ Hz. This is known as dielectric relaxation^19–21^. The dielectric relaxation observed at higher frequencies could be caused by charge carrier resonance within the grain. Consequently, the tanδ dramatically increases. The microstructural results indicate that the increased tanδ of the NY15h ceramic compared to NY9h may be due to a reduction in the overall resistance of a material, particularly the GB response. Using a Z* plot, it is possible to estimate the resistances of gains (*R*_g_) and GBs (*R*_gb_). The wide semicircular arc represents the electrical response of GBs, while the nonzero intercept represents the electrical response of the grains. Although *R*_gb_ cannot be determined at this temperature, as indicated in Fig. 4c, these Z* spectra may be used to infer that the *R*_gb_ of NY9h ceramic is greater than that of the NY15h ceramic by comparing the slopes of these plots. The Z* plots of NY9h and NY15h at 60 °C are shown in the inset of Fig.4c. It was clearly seen that tendencies of the Z* plot at temperatures of 20 °C and 60 °C are identical. A greater *R*_gb_ value for the NY9h ceramic is strongly related to its lower tanδ in the frequency range below 10^3^ Hz compared to the NY15h ceramic. The increase in tanδ in the low-frequency range of the NY15 ceramic is directly related to greater DC conduction caused by charge migration over long distances^10,11^, as shown by Eq. (3):

3 $$\tan \delta \approx \frac{{\sigma_{dc} }}{{\omega \varepsilon_{0} \varepsilon_{s}^{\prime } }},$$

where *σ*_dc_ denotes DC conductivity and ε′_s_ is the dielectric permittivity at low frequencies. At low frequencies, it was discovered that the connection between DC conductivity and the tanδ closely follows Eq. (3). The tanδ values at 1 kHz of NY9h and NY15h were 0.035 and 0.032, respectively. It was found that the ε′ values of NY9h and NY15h are greater than those reported for NYCTO ceramics prepared using the SSR^19^ and spark plasma sintering methods^20^. Additionally, tanδ of our samples is smaller than that of the NYCTO ceramics synthesized by both SSR and spark plasma sintering approaches^19,20^. Hence, dielectric property of our NYCTO ceramics is superior to that of the NYCTO ceramics obtained by the SSR and spark plasma sintering methods.

With decreased sintering temperature, retention of high ε′ values and reduction of tanδ were significant accomplishments in the production and study of the properties of NYCTO ceramics using a modified sol-gel method. Given the equation, *R*_gb_=1/ω*C*_gb_, where *C*_gb_ is the GB capacitance, it is plausible to suggest that the increase in the ε′, in addition to being caused by increased grain sizes, may also result from a reduction in *R*_gb_ compared to the NY9h specimen. The nonzero intercept showing the electrical response inside the grains was also seen in Z* plots [figure not shown]. The estimated *R*_g_ values of the NY9h and NY15h ceramics are 98 and 103 Ω⋅cm, respectively, as listed in Table 3. Although the sintering times for the NY9h and NY15h ceramics vary by approximately 6 h, their *R*_g_ values are almost identical.

**Table 3:** *ε*′ and tanδ at 1 kHz and 20 °C, *R*_g_ at 20 °C, *R*_gb_ at 60 °C, activation energy of relaxation (*E*_a_), and activation energies of grains (*E*_g_) GBs (*E*_gb_), breakdown electric field (*E*_b_), and nonlinear coefficient (α) of the NY9h and NY15h ceramics.

Samples	ε′	tanδ	*R*_g_ (Ω.cm)	*R*_gb_ (Ω.cm)	*E*_a_ (eV)	*E*_g_ (eV)	*E*_gb_ (eV)	*E*_b_ (V/cm)	α
NY9h	1.37×10^4^	0.035	98	1.77×10^6^	0.138	0.114	0.717	6.28×10^3^	7.83
NY15h	1.99×10^4^	0.032	103	3.32×10^5^	0.113	0.104	0.674	5.83×10^3^	7.89

**Figure 5:**
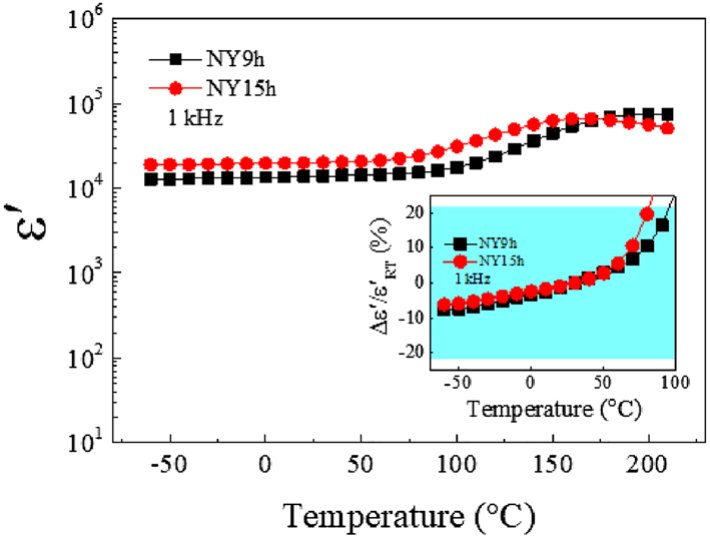
Temperature dependence of ε′ at 1 kHz of NY9h and NY15h ceramics. Its inset shows the temperature coefficient of ε′ at 1 kHz.

Figure 5 depicts the temperature dependencies of ε′ at 1 kHz for NY9h and NY15h ceramics. The ε′ values of both NY9h and NY15h ceramics are likely stable at temperatures below 90 °C. Additionally, ε′ slightly increased at temperatures greater than 90 °C. This indicates that DC conduction predominates in NYCTO ceramics^10,11^. Such dielectric behavior is similar to that reported in Na_1/2_Sm_1/2_Cu_3_Ti_4_O_12_ ceramics^30^. In the current work, the temperature coefficients of ε′ at 1 kHz for the NY9h and NY15h ceramics were evaluated. At temperatures ranging from − 60 to 80 °C, the ε′ values of both NY9h and NY15h ceramics changed less than 22% in comparison to ε′ at room temperature. It is well known that preparation method directly affects the physical properties such as lattice parameters, grain size, electrical and dielectric properties. According to the requirements for commercial ceramic capacitors, ε′, tanδ, and temperature stability of the ε′ value are vital parameters^14^ i.e. a loss tangent less than 0.05 at 1000±50 Hz and room temperature, and dielectric permittivity greater than 5000. In the present work, the NYCTO ceramics prepared using a modified sol-gel method give very high ε′ (1.37–1.55×10^4^), whereas the ε′ of NYCTO ceramics obtained by the SSR and spark plasma sintering methods ranges from 10^3^ – 10^4^. Moreover, we would like to emphasize that the ε′ of our NYCTO ceramics is also 2 – 11 times higher than that of Y_2/3_Cu_3_Ti_4_O_12_^31^ fabricated by sol-gel approach, Na_1/2_Sm_1/2_Cu_3_Ti_4_O_12_^32^ and Na_1/2_La_1/2_Cu_3_Ti_4_O_12_^33^ produced by the SSR method. Comparing the tanδ, it was found that tanδ values of our synthesized ceramics (0.032–0.035) are indeed low in comparison to the tanδ of the NYCTO obtained from other preparation methods^19,20^. Additionally, tanδ of Y_2/3_Cu_3_Ti_4_O_12_^31^, Na_1/2_Sm_1/2_Cu_3_Ti_4_O_12_^32^ and Na_1/2_La_1/2_Cu_3_Ti_4_O_12_^33^ ceramics is larger than our obtained tanδ. According to Fig. 5, the temperature coefficients of ε′ at 1 kHz of both NY9h and NY15h ceramics change less than 22% at temperatures ranging from − 60 to 80 °C. Based on these results, the ε′ and tanδ values, as well as the temperature coefficients of ε′ value of our NYCTO meet the standard of Class III capacitors, such as X5U and Y5U capacitors^14^.

**Figure 6:**
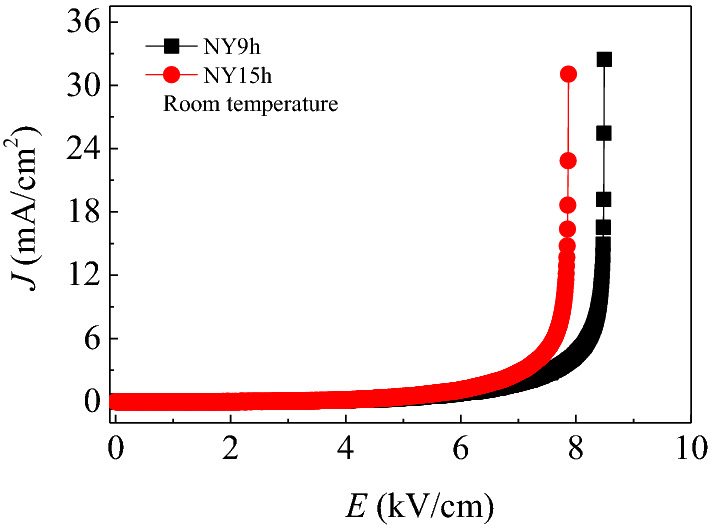
Nonlinear J – E properties of NY9h and NY15h ceramics at room temperature.

The nonlinear J-E characteristics of sintered NYCTO at room temperature are illustrated in Fig. 6. We found that the relationship between J and E is nonlinear resulting from the presence of an IBLC structure in these specimens. This observation is in excellent agreement with Chung et al.^26^. In the current work, the breakdown electric field (*E*_b_) and the nonlinear coefficients (α) of the NY9h and NY15h ceramics were computed. The *E*_b_ values were 6.28×10^3^ and 5.83×10^3^ V/cm for NY9h and NY15h ceramics, respectively, as displayed in Table 3. By increasing the sintering time from 9 to 15 h, the reduction of *E*_b_ correlates to a reduction in the *R*_gb_ of these ceramics. The α values of the NY9h and NY15h ceramics were 7.83 and 7.89, respectively.

**Figure 7:**
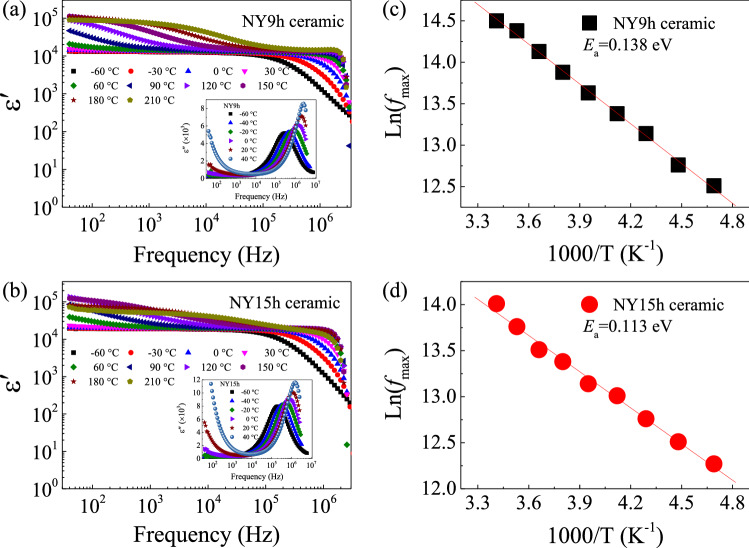
(**a**,**b**) Frequency dependencies of ε′ at various temperatures of NY9h and NY15h ceramics, respectively. Inset of each figure discloses the frequency dependency of ε″. (**c**,**d**) Arrhenius plots of *f*_max_ of these ceramic samples.

To investigate the dielectric properties of NY9h and NY15h ceramics as a function of temperature, the frequency dependencies of the ε′ and ε″ of these two specimens at different temperatures are shown in Fig. 7a,b and insets. At frequencies less than 10^5^ Hz, the ε′ values tend to increase with temperature. ε″ also increases with temperature over the same frequency range. The dielectric behavior in this frequency range may be attributed to DC conduction induced at GBs and other factors, such as the sample-electrode contact^34^. At frequencies greater than 10^5^ Hz, a fast reduction of ε′ is observed, which corresponds to the rapid increase in the ε″ value at the same frequencies. Additionally, these reduced ε′ values move to higher frequencies when temperature is increased. This dielectric behavior is generated inside the grains as a result of the presence of oxygen vacancies, which is often referred to as the dielectric relaxation process^21^. As illustrated in Fig. 7a and b, thermal energy can activate a dielectric relaxation process, as shown by the peak position of ε″ shifting to a higher frequencies as temperature increases^21^. The activation energy for high-frequency relaxation (*E*_a_) was determined. Temperature dependency of frequency at the maximum-ε″ peak (*f*_max_) is expressed as:

4 $$f_{\max } = f_{0} \exp \left( {\frac{{ - E_{a} }}{{k_{B} T}}} \right),$$

where *f*_0_ denotes a constant term and *k*_B_ is the Boltzmann constant. T is the absolute temperature. As shown in Fig. 7c and d, the temperature dependence of *f*_max_ can be well fitted by Eq. (4). The slope of the fitted lines is used to calculate *E*_a_, and the resulting values are shown in Table 3. *E*_a_ values for the NY9h and NY15h ceramics were found to be 0.138 and 0.113 eV, respectively. These *E*_a_ values are quite close to those reported by Liang et al.^27^. It is established that the first  (V_o_^+^) and second ionization  (V_o_^++^) of oxygen vacancies may be readily produced through a procedure such as high-temperature sintering^35^. For instance, when CCTO is heated, intergranular oxygen is liberated from the lattice, resulting in the creation of oxygen vacancies (V_o_). During the cooling process, O^2-^ is used to fill some V_o_ sites, particularly those in the GB layers. As a consequence, V_o_ is detected within the grains^35^.

Due to the inherent defects of V_o_^+^ and V_o_^++^, donor energy levels ranging from 0.01–0.07 eV and 0.10–0.20 eV, respectively, are produced^27^. It is possible that *E*_a_ values ranging from 0.113–0.138 eV arose from the electrical response inside the grains of the NY9h and NY15h ceramics as a result of the presence of V_o_^++^ in the ceramics. The steps of dielectric relaxation in a high-frequency region of each sample are strongly linked to their *E*_a_ values.

The activation energy of conduction for grains (*E*_g_) and GBs (*E*_gb_) can be calculated using the Arrhenius law for resistances,

5 $$R_{g,gb} = R_{0} \exp \left( {\frac{{E_{g,gb} }}{{k_{B} T}}} \right),$$

where *R*_0_ is the pre−exponential constant term. In the current study, *R*_gb_ cannot be calculated from the Z* spectra of the NY9h and NY15h ceramics due to the dominant effect of sample-electrode contact in the low-frequency region. A complex electric modulus (M*=M′+jM″) was considered to eliminate the effect of a sample-electrode contact from the *R*_gb_ calculation. The following equations can be used to construct an M* plot:

6 $$M^* = j\omega C_{0} Z^* = M^{\prime} + jM^{\prime\prime},$$

7 $$M^{\prime\prime}_{\max } = C_{0} /2C_{gb} ,$$

8 $$\omega \tau_{gb} = \omega R_{gb} C_{gb} = 1.$$

**Figure 8:**
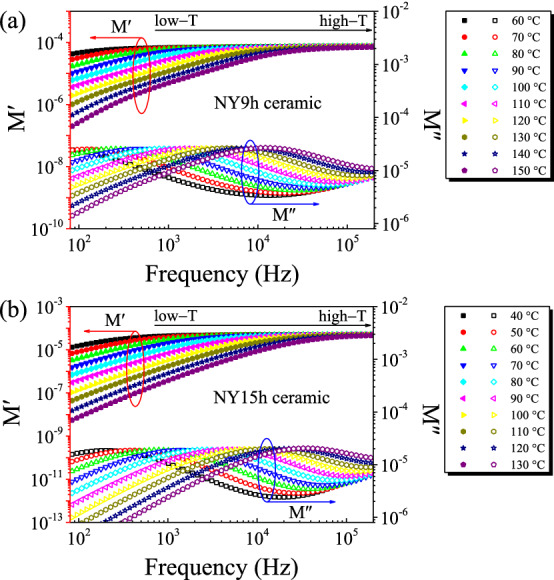
Frequency dependencies of M′ and M″ at various temperatures for (**a**) NY9h and (**b**) NY15h ceramics.

M″_max_ represents the maximum value of M″ and τ_gb_=1/ω_max_ denotes the variation of relaxation time. Fig. 8 depicts the frequency dependencies of M′ and M″ in sintered NYCTO samples. The patterns for the shift in the M″_max_ position were comparable for NY9h and NY15h ceramics. The M″_max_ location quickly shifted to higher frequencies with increased temperature, demonstrating thermally activated dielectric relaxation^21^. Furthermore, this pattern indicated a reduction in *R*_gb_ as a function of increasing temperature. The *R*_gb_ values of the NY9h and NY15h samples at 60 °C were estimated using M* plots and found to be 1.77×10^6^ and 3.32×10^5^ Ω⋅cm, respectively. The *R*_gb_ values of each sample are consistent with the tanδ in the low-frequency range.

**Figure 9:**
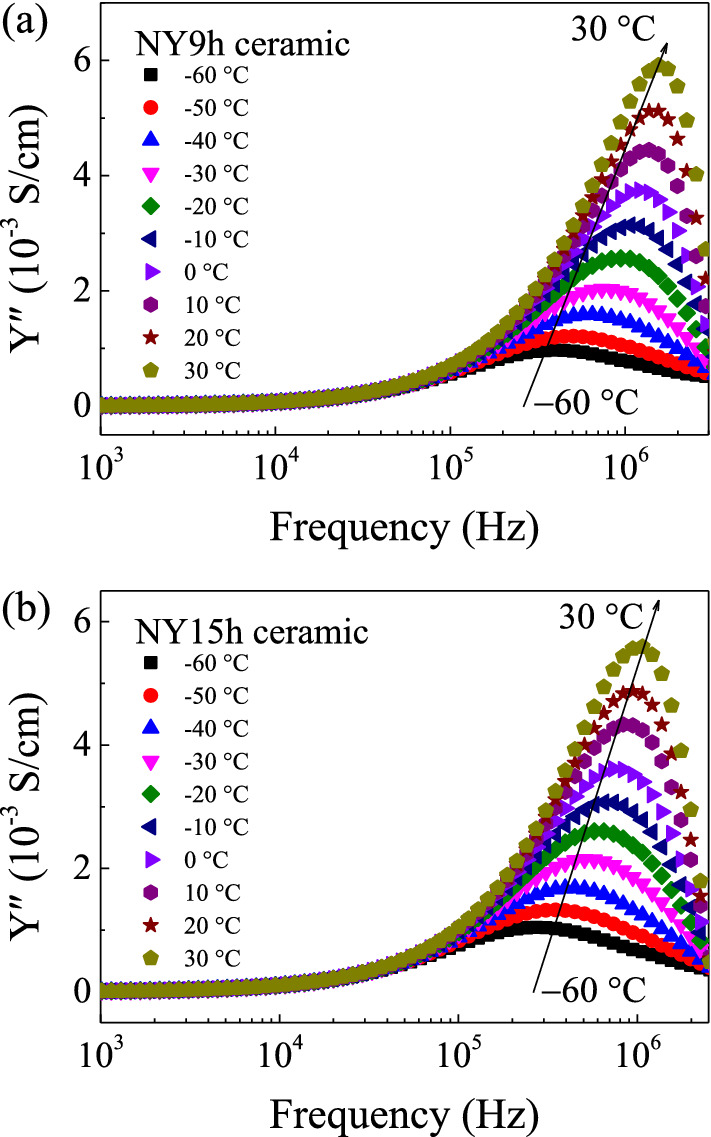
Frequency dependency of Y″ at various temperatures for (**a**) NY9h and (**b**) NY15h ceramics.

Additionally, the temperature dependency of *R*_g_ was investigated using complex admittance (Y*=Y′+jY″),

9 $$Y^* = Y^{\prime} + jY^{\prime\prime} = \frac{1}{{Z^{ * } }},$$

where Y′ and Y′′ represent the real and imaginary parts of Y*, respectively. In general, *R*_g_ values can be approximated from the nonzero intercept of Z* plots. *R*_g_ may also be calculated *via* admittance spectroscopy (AS) analysis using the equation, *R*_g_ = 1/2Y″_max_, where Y″_max_ is the highest value at the Y′′ peak. Y′′ peaks were observed in the frequency range, 10^5^–10^6^ Hz, for the NY9h and NY15h ceramics at temperatures below 30 °C, as shown in Fig. 9a and b. The magnitude of the Y′′ peak increased with temperature, suggesting a reduction in *R*_g_. *R*_g_ and *R*_gb_ are temperature-dependent, as seen in the M* and Y* plots.

**Figure 10:**
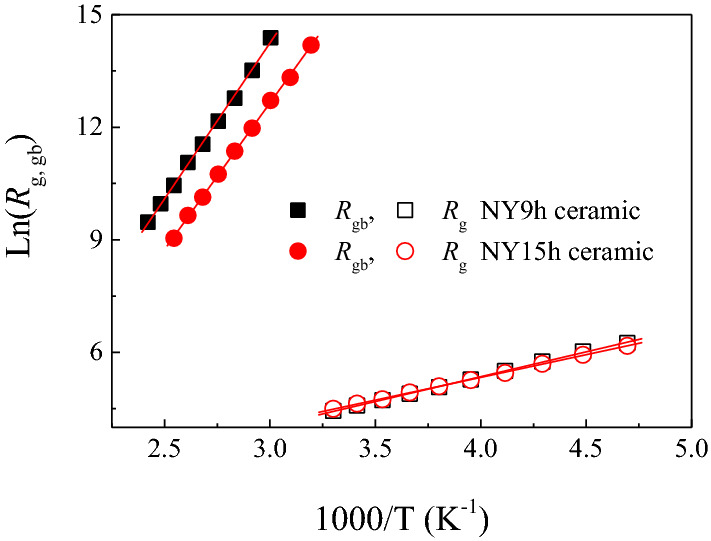
Arrhenius plots of *R*_g_ (open symbols) and *R*_gb_ (solid symbols) for NY9h and NY15h ceramics.

As illustrated in Fig. 10, *R*_gb_ and *R*_g_ variation followed the Arrhenius law for all samples. *E*_g_ and *E*_gb_ values can be estimated from the slopes of linearly fitted curves. *E*_g_ values for the NY9h and NY15h ceramics were 0.114 and 0.104 eV, respectively. The patterns of *E*_g_ and *E*_a_ changes are similar in NY9h and NY15h ceramics, demonstrating the electrical response inside the grains of these two samples. The *E*_gb_ values calculated from the Arrhenius equation for NY9h and NY15h ceramics were 0.707 and 0.674 eV, respectively. The difference in the values of *E*_g_ and *E*_gb_ of more than 0.5 eV indicates a heterogeneous microstructure with semiconducting and insulating components. This observation is quite similar to the IBLC model for dielectrics^35^. The *E*_gb_ and *R*_gb_ values of the NY9h and NY15h ceramics are consistent with their low-frequency tanδ values.

**Figure 11:**
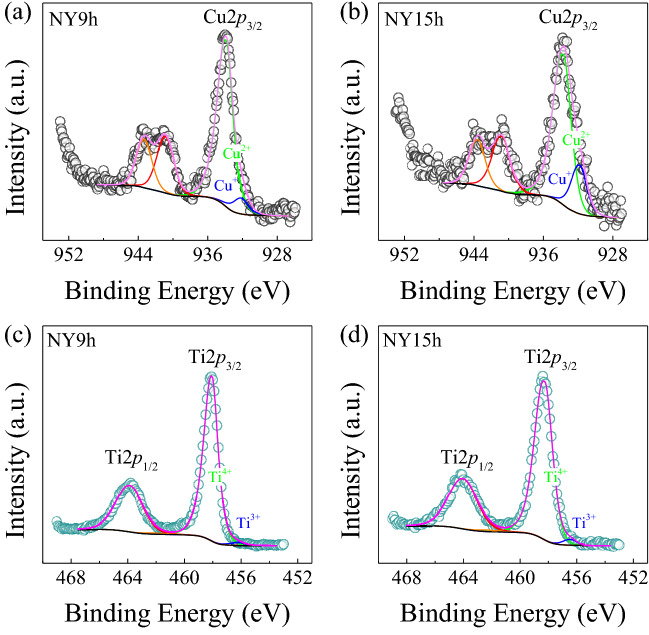
(**a**,**b**) XPS Cu2*p* spectra of NY9h and NY15h ceramics. (**c**,**d**) XPS Ti2*p* spectra of these two samples.

The electronic structures of transition ions were also investigated in this research. The XPS technique was used to thoroughly examine the valence states of Cu and Ti ions. XPS Cu2*p* spectra were obtained in the binding energy (BE) range of 926-969 eV, whereas the XPS Ti2*p* spectra were recorded in the BE range of 448–469 eV. Fig. 11 shows the XPS Cu2*p* and Ti2*p* spectra. Overlapping peaks are seen in the Cu2*p*_3/2_ peak, as illustrated in Figs. 11a and b. The main peaks were found at BE positions of ∼933.88 and ∼933.69 eV for NY9h and NY15h samples, respectively. Minor peaks in the Cu2*p*_3/2_ peak of these two samples were seen at BE positions of 932.06 and 931.81 eV, respectively. The BE difference between the main and minor Cu2*p*_3/2_ peaks is about 1.82-1.88 eV. The main and minor peaks indicate the presence of Cu^2+^ and Cu^+^ in both NY9h and NY15h structures. The ratio of Cu^+^/Cu^2+^ for NY9h and NY15h was 8.47/91.53 and 26.36/73.64%, respectively. An overlapping peak was also seen in the XPS Ti2*p* spectra. Significant Ti^4+^ peaks were detected at BE positions of 458.05 and 458.26 eV for NY9h and NY15h samples, respectively. Very small Ti^3+^ peak differences were identified in the NY9h and NY15h structures of approximately 1.83–1.90 eV. These peaks were observed at BE positions of 456.15 and 456.43 eV, respectively. The Ti^3+^/Ti^4+^ ratio was 2.37/97.63 and 2.69/97.31% for NY9h and NY15h, respectively. The presence of Cu^+^ and Ti^3+^ is in agreement with earlier literature reports^30,34^. Cu^+^ and Ti^3+^ in the NYCTO lattice indicates the presence of V_O_ in the structure. Due to charge balances, the loss of the lattice oxygen (O_L_) may cause Cu and Ti ions to reduce to their oxidation states. As a consequence of the charge compensation mechanism, it is plausible that creation of *n*-type semiconducting grains in sintered NYCTO ceramics may be due to charge carrier hopping between Cu^+^↔Cu^2+^ and Ti^3+^↔Ti^4+^, generating a small level of conductivity inside the grains of this ceramic.

According to Table 2, the *ɑ* values of both NY9h and NY15h ceramics are lower than those of CCTO^28^. This is due to the difference in sizes of both Na and Y in comparison to Ca and Cu. From the experimental investigations of Shannon^36^ and Tkach et al.^37^, the covalent radii of Y^3+^ with 9 coordination numbers, Ca^2+^ with 12 coordination numbers and Cu^2+^ with 6 nearest neighbors are 1.23 Å, 1.48 Å and 0.87 Å, respectively. In the case of Y-doped CCTO, earlier experimental investigations^38,39^ revealed that the lattice constant of this structure is reduced. In other words, Y ions should occupy sites with larger covalent radii. Due to the smaller covalent radius of Y^3+^ compared to Ca^2+^, the Y^3+^ ions preferentially occupy Ca sites.

Next, the occupation site of Na in the CCTO host is determined. As discussed above, substitution of Y^3+^ at Ca cites leads to a decreased lattice constant. From Table 2, the lattice parameter of the NY9h and NY15h samples is smaller than that of the intrinsic CCTO. It is impossible for Na^+^ to occupy sites with a larger covalent radius, namely Ca^2+^ sites, because the lattice parameter would be reduced, resulting in a large decrease in the lattice constant, which is in conflict with our XRD measurements. Therefore, Na^+^ must occupy sites with smaller covalent radii. It was found that the covalent radii of Na^+^ and Cu^2+^ with 6 nearest neighbors are 1.16 and 0.87 Å, respectively^36^. Hence, Na^+^ is likely to occupy Cu^2+^ sites. This observation is confirmed by earlier experimental and theoretical investigations^34,40–42^. As demonstrated by Li et al.^43^, Ti^4+^ ions can reduce their oxidation state to Ti^3+^. Then, the Ti^3+^ ions occupy the Cu^2+^ sites in the CCTO structure. For another perovskite ceramic, specifically SrCu_3_Ti_4_O_12_^44^, it was found that Ti ions can also occupy Cu sites in this structure, resulting in a release of Cu ions from this lattice. Similarly, in our work, Cu^2+^ sites are occupied by Na^+^ ions in the NYCTO ceramics leading to our observation of excess Cu at the grain boundary, as can be clearly seen in Fig. 3a. The substitution of Na atoms at Cu sites is described by the following reaction:

$$2Na + CuO + \frac{1}{2}O_{2} \mathop{\longrightarrow}\limits^{2CuO}2Na^{\prime}_{Cu} + Cu + 2O_{O} .$$

In our experiment, we fabricated Na_1/2_Y_1/2_Cu_3_Ti_4_O_12_ based on the assumption that Na and Y ions occupy Ca sites. The EDS mapping presented in Fig. 3a reveals that Cu-rich phases (green color) are clearly present at the GB layers of this ceramic.

**Figure 12:**
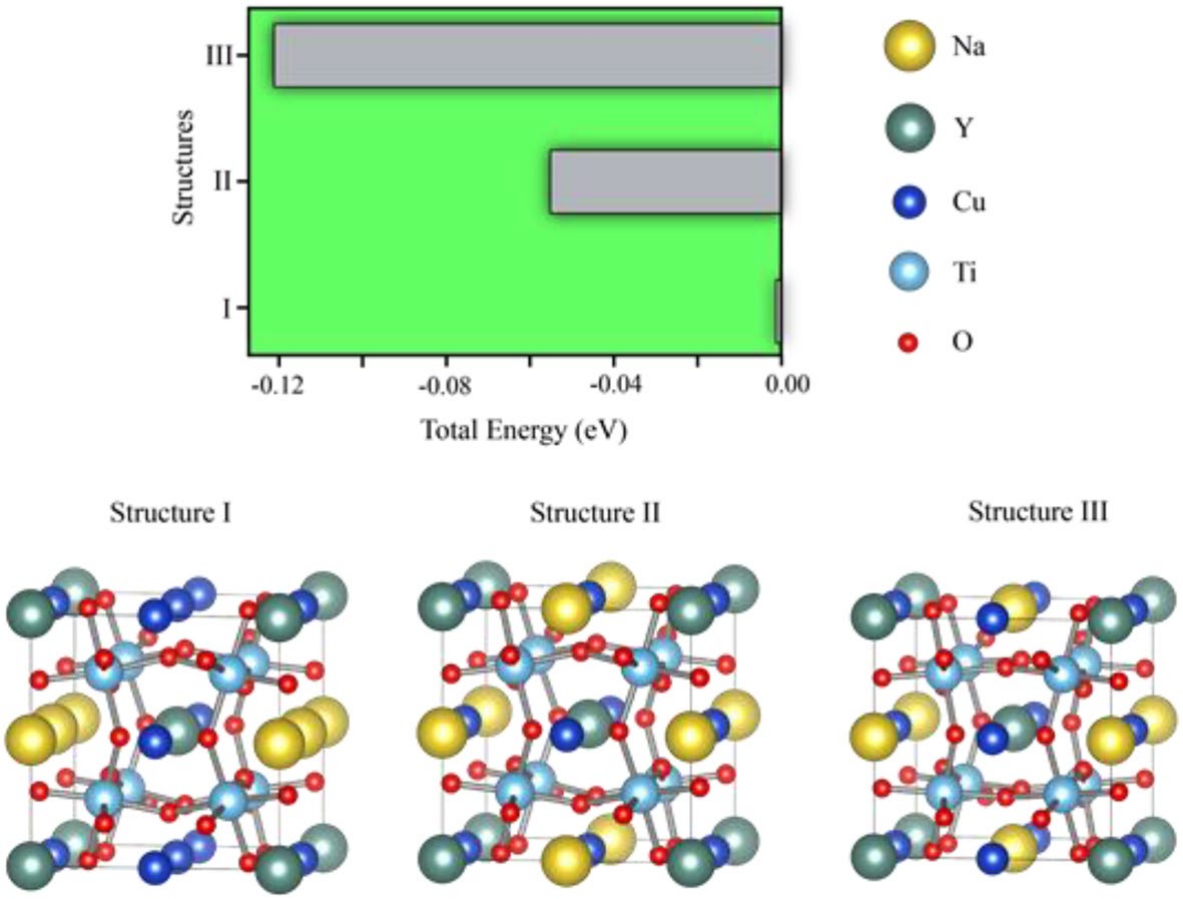
Total energy of three different configurations of Na and Y atoms in the CCTO lattice.

This observation was associated with substitution of either Na or Y dopants at the Cu sites. Based on our previous results, Na and Y ions occupy Cu and Ca sites, respectively, and the chemical formula of the NYCTO used in our calculations is Na_2_Y_2_Cu_4_Ti_8_O_24_. Three possible configurations, namely Structures I–III of Fig. 12, are considered to determine the most stable structure of this NYCTO ceramic. As presented in Fig. 12, Structure III gives the lowest total energy making it the most stable. Also, in the present work, we calculated the electron density difference between NYCTO and CCTO ($\Delta \rho_{A} ({\text{r}})$) which is given by:

10 $$\Delta \rho_{A} ({\text{r}}) = \rho_{NYCTO} ({\text{r}}) - \rho_{CCTO} ({\text{r}}).$$

**Figure 13:**
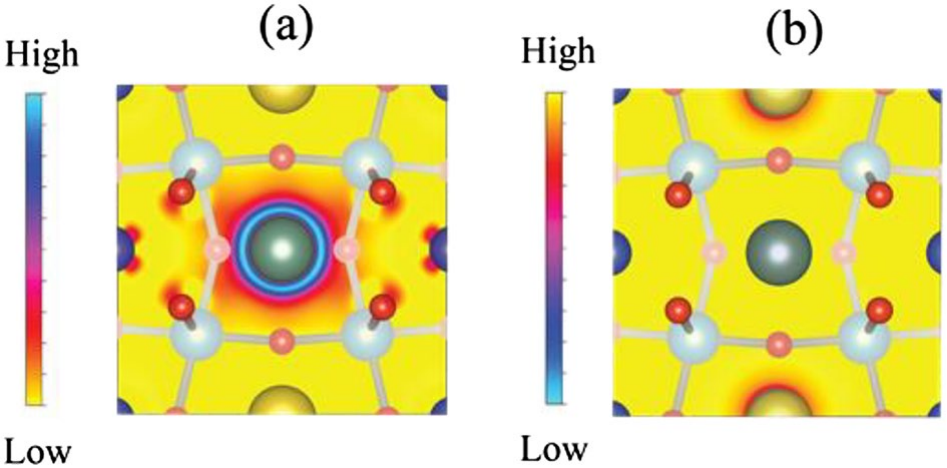
(**a**) and (**b**) the positive and negative electron-density differences of the (100) plane between NYCTO and CCTO, respectively.

Here, $\rho_{CCTO} ({\text{r}})$ and $\rho_{NYCTO} ({\text{r}})$ represent the electron density of CCTO and NYCTO structures, respectively. From Eq. (10), the region with $\Delta \rho_{A} ({\text{r}}) > 0$ corresponds to a region of electron accumulation. Conversely, electron depletion occurs in regions with negative $\Delta \rho_{A} ({\text{r}})$ values. Regions with positive and negative $\Delta \rho_{A} ({\text{r}})$ values are presented in Fig. 13a and b, respectively.

**Figure 14:**
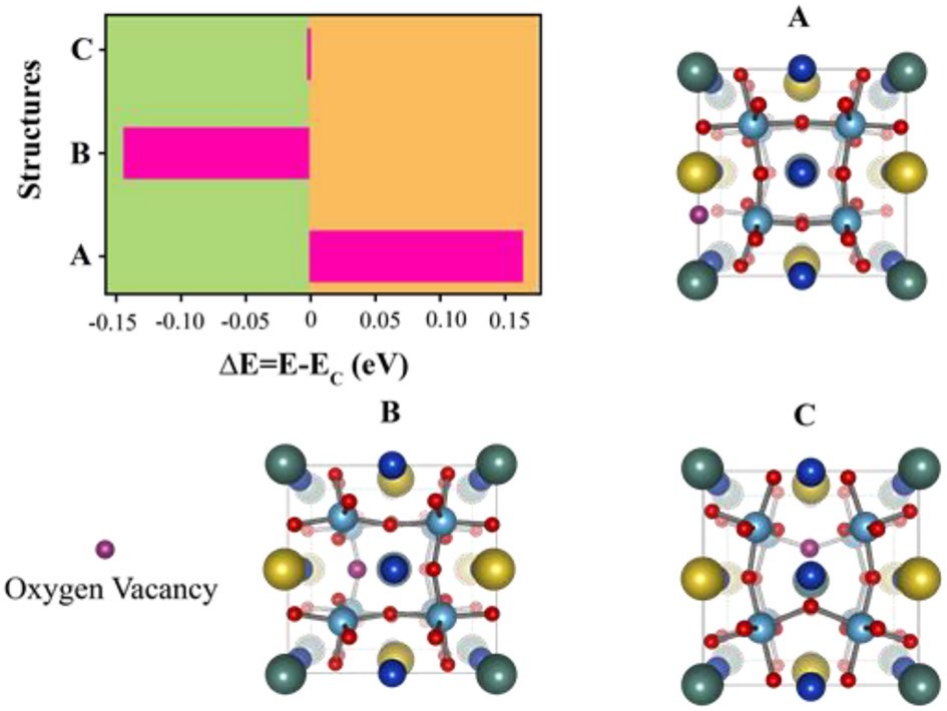
Total energy of various positions of oxygen vacancy in the NYTO lattice in comparison to total energy of Structure C. Structure A is a structure with an oxygen vacancy between Na and Y ions. For Structure B, the oxygen vacancy is coupled with both Na and Cu in the NYCTO structure. V_o_ is close to Y labelled in Structure C.

In Fig. 13a, the electron density on a Y atom (green ball) is indeed high. Hence, there is an electron accumulation on Y ions resulting in less positively charged Y ions. In Fig. 13b, the electron depletion region is only found at Na atoms (yellow balls). This results in a more positive valence state for Na in the NYCTO. Based on our electron density calculations, the Y atom becomes less positive, whereas the Na valence state is more positive. Consequently, there is a charge compensation between Na and Y in the NYCTO ceramic when both Na and Y are co-doped into the CCTO structure. Experimentally, V_o_ are always observed during a sintering process. The most stable position of Vo in the NYCTO needs to be clarified. Using Structure III, presented in Fig. 12 as an initial structure, only three possible locations of V_o_ in this host are considered. For Structure A, a V_o_ is located between the Na and Y ions. Positioning V_o_ between Na and Cu atoms is presented as Structure B. In Structure C, the V_o_ is in close proximity to Y. The total energy corresponding to these three structures is given in Fig. 14. Based on our total energy results, we found that total energy of Structure B is the lowest followed by Structures C and A, in that order. Consequently, V_o_ is likely between the Na and Cu atoms in an NYCTO host. As shown in Fig. 14, it is reasonable to suggest that Na and Cu atoms in NYCTO can preferentially induce V_o_ during a sintering process at high temperatures. In this structure, we found that V_o_ is also in close proximity to Ti atoms. This observation might be related to the Cu^+^ and Ti^3+^ in our samples.

To investigate the origin of the Cu^+^ and Ti^3+^ in the NYCTO ceramic, the electron density difference of NYCTO with and without V_o_ ($\Delta \rho_{B} ({\text{r}})$) was determined. The $\Delta \rho_{B} ({\text{r}})$ values can be evaluated using the following equation.

11 $$\Delta \rho_{B} ({\text{r}}) = \rho_{NYCTO + V_o} ({\text{r}}) - \rho_{NYCTO} ({\text{r}}).$$

**Figure 15:**
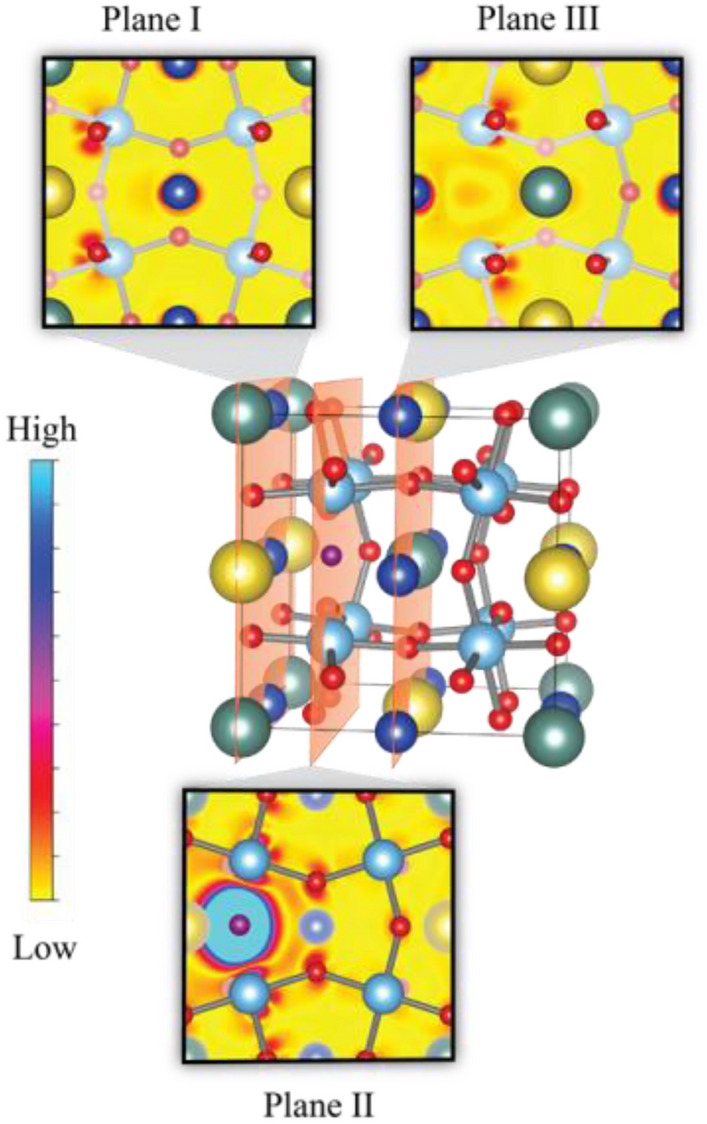
Electron density difference between NYCTO with and without oxygen vacancies of three planes, namely, planes I–III.

$\rho_{NYCTO + V_o} ({\text{r}})$ is the electron density of Structure B, as presented in Fig. 14. The calculated $\Delta \rho_{B} ({\text{r}})$ is illustrated in Fig. 15. In this figure, we considered the $\Delta \rho_{B} ({\text{r}})$ values at three (100) planes of the NYCTO +V_o_ structure. It can be seen from Fig. 15 that the electron density on Cu (dark blue balls) and Ti (light blue ball) gives a positive value for $\Delta \rho_{B} ({\text{r}})$, although, the electron density on both Na and Y is unchanged. So, Cu^2+^ and Ti^4+^ should be reduced to Cu^+^ and Ti^3+^, respectively. In other words, the Cu^+^ and Ti^3+^ ions observed in our XPS measurements (Fig. 11) originate from V_o_ in the NYCTO lattice.

In general, the small conductivity in oxides containing V_o_ can be attributed to electronic charge hopping between similar ions. Additionally, in related NYCTO structures, such as CaCu_3_Ti_4_O_12_, phase transition does not occur when the temperature increases, as reported by Subramanian et al.^28^. So, in this case, the ferroelectric effect can be ignored. For NYCTO ceramics, a small conductivity (within the grains) due to electron hopping between Cu^+^↔Cu^2+^ and Ti^3+^↔Ti^4+^ might play an important role in the colossal dielectric response of this material. According to previous studies, variation of dielectric response can be controlled by metal-ion doping^34,42^ in the ceramic lattice or annealing at various atmospheres^45^.

These conditions can induce formation of V_o_. Consequently, it is reasonable to suggest that the formation of V_o_ in the lattice is crucial to produce the colossal dielectric response in NYCTO ceramics. This finding supports the hypothesis that the IBLC effect is the primary origin of the dielectric response of NYCTO ceramics.

## Conclusions

Both sintered Na_1/2_Y_1/2_Cu_3_Ti_4_O_12_ ceramics produced using a modified sol-gel method contain a CaCu_3_Ti_4_O_12_ primary structure. Colossal dielectric properties were acquired by sintering at a low temperature. Na_1/2_Y_1/2_Cu_3_Ti_4_O_12_ ceramics sintered at 1050 °C for 9 and 15 h give very high dielectric permittivities of 1.37–1.99×10^4^ with a low loss tangent, less than 0.04. According to the DFT investigations, Na ions are more likely to occupy Cu sites and Ca sites host Y ions. Related Cu phases, particularly CuO, were discovered near grain boundaries due to the substitution of Na ions at Cu sites. These related Cu phases forming as layers with high resistance result in enhanced grain boundary response. Consequently, electrical performance of these ceramics is improved. Based on electron density calculations, we found that trend of the Na charge is opposite to the trend of Y charge, leading to a charge compensation mechanism in Na_1/2_Y_1/2_Cu_3_Ti_4_O_12_. Moreover, the Cu^+^ and Ti^3+^ states in the sample are from the presence of oxygen vacancies in the lattice. Finally, an internal barrier layer capacitor may be the primary origin of the colossal dielectric response in these ceramics.

## Methods

### Gel and ceramic preparations

C_2_H_3_O_2_Na (Sigma−Aldrich, 99.995% purity), C_6_H_9_O_6_Y·H_2_O (Sigma−Aldrich, 99.9% purity), C_4_H_6_CuO_4_⋅H_2_O (Sigma−Aldrich, 99.0% purity), C_16_H_28_O_6_Ti (Aldrich, 75 wt. % in isopropanol), C_6_H_8_O_7_⋅H_2_O (RCL Labscan, 99.5% purity), C_2_H_6_O_2_ (QRëC, 99.5% purity), C_2_H_5_OH (RCL Labscan, 99.5% purity), and de-ionized water were the raw materials used in the preparation process of Na_1/2_Y_1/2_Cu_3_Ti_4_O_12_ *via* a modified sol-gel technique. Details of the synthesis, beginning with gel preparation and ending with a mixed precursor before calcination, have previously been reported^46^. The resulting powder was calcined in air at 900 °C for 12 h before being ground in a mortar and pestle to yield a fine calcined powder. Under uniaxial compression at 200 MPa, the NYCTO powder was shaped into green bodies that were 9.5 mm in diameter and 2 mm thick. The green bodies of NYCTO powders were sintered in air for 9 (NY9h) and 15 h (NY15h) at 1050 °C. The sintering temperature in the current work is approximately 50 °C lower than that of ordinary solid-state reaction methods. The heating rate during the sintering process is 5 °C /min. Once the furnace is shut off, the material is cooled to room temperature by natural cooling.

### Characterizations

X-ray diffractometer (XRD, PANalytical, EMPYREAN) was used to examine the phase composition and crystal structure of sintered materials. The 2θ range utilized in XRD data collection was from 20 to 80°. A step increment of 0.01 degree/point was used for XRD data collection. The Rietveld refinement method was employed to analyze the XRD data. Zero shift, scale factor, background (with a polynomial function type), profile half-width parameters (v, u, w), lattice parameters (a, b, c), atomic site occupancies (Wyckoff), preferred orientation parameter, and site occupancy fraction (SOF) were the parameters and coefficients used for optimization in Rietveld refinement. The surface microstructure of NY9h and NY15h ceramics was studied using desktop scanning electron microscopy (SEM, SEC, SNE-4500M). The accelerating voltage for SEM measurements was 20 kV. Before analyzing the bulk microstructure, the cross-sections of all NYCTO ceramics were polished using a diamond polishing pad, at a rotation rate of 300 rpm, until the cross-sectional layer was smooth. The ceramics were then annealed in air for 5 min at 1010 °C. Field Emission Scanning Electron Microscopy (FE-SEM, FEI) operating in the energy dispersive X-Ray spectroscopy (EDS) mode was utilized to examine the elemental distributions of sintered samples. The relative density (*D*) was analyzed using the Archimedes method. ImageJ software was also used to estimate grain sizes and size distributions. X-ray photoelectron spectroscopy (XPS, AXIS Ultra DLD) was employed to investigate the oxidation states of transition elements. The XPS data were processed using MultiPak software, which is based on a Gaussian-Lorentzian profile fit.

### Dielectric testing

Before testing, both the NY9h and NY15h ceramics were polished to obtain clean and flat surfaces. Then, Au was coated to a thickness ~40 nm on both parallel surfaces of these two samples using a Polaron SC500 sputter coater. The dielectric properties of the NY9h and NY15h ceramics were measured using a KEYSIGHT E4990A analyzer with a 0.5 V of oscillation voltage (V_rms_). The stability of dielectric properties was investigated across frequency and temperature ranges of 40 to 10^7^ Hz and − 60 to 210 °C, respectively. A temperature controller (9023 Delta Design Chamber) was utilized to control the measurement temperature with increasing the temperature in 10 °C increments. The nonlinear J – E properties were evaluated at room temperature (RT) utilizing a high voltage measurement unit (Keithley Model 247) coupled to a programmable electrometer (Keithley Model 617). In our J – E measurements, the load frequency for applied voltage is set to be 0.95 V/s.

### DFT calculations

Electronic structure and electron density calculations of NYCTO ceramics were computed using the Vienna *Ab initio* Simulation Package (VASP)^47^. The pseudopotential used in this work is based on the Projector Augmented Wave technique. The Perdew–Burke–Ernzerhof (PBE) form of the exchange-correlation potential^48^ was employed. Valence states of Cu, Ti and O were obtained from published literature^42^. The valence states of Na are 2s, 2p and 3s. Moreover, 4s, 4p, 5s and 4d were chosen as the valence states of Y. According to the total energy convergence tests, 650 eV of plane wave cutoff energy and 7×7×7 k-point sampling of the reciprocal spaces were observed. The conjugate-gradient technique was employed to relax the NYCTO structures.

## Data Availability

The data of this study are available from the corresponding author upon reasonable request.
